# High-grade desmoplastic infantile astrocytoma in a 1-year-old child with Down’s syndrome: a case report

**DOI:** 10.1186/s13256-022-03615-0

**Published:** 2022-11-04

**Authors:** Muhammad Hamza Habib, Mehvish Zahra Alavi, Amber Goraya, Samina Zaman, Alia Ahmed

**Affiliations:** 1 Rutgers Robert Wood Johnson School of Medicine, Cancer Institute of New Jersey, 195 Little Albany Street, New Brunswick, NJ 08901 US; 2 The Children’s Hospital and Institute for Child Health. Ferozepur Rd, Nishtar Town, Lahore, Pakistan

**Keywords:** Down’s syndrome, Desmoplastic infantile astrocytoma, Desmoplastic infantile ganglioglioma, Childhood brain tumors

## Abstract

**Background:**

Down’s syndrome is the most common chromosomal abnormality in humans. It has been associated with central nervous system tumors such as primary acute lymphoblastic leukemia and germinomas, but desmoplastic infantile astrocytoma has not yet been reported with Down’s syndrome. Desmoplastic infantile astrocytoma is a rare intracranial tumor that mostly occurs in the first 2 years of life. It usually presents as a large, aggressive tumor with both solid and cystic components. Genetically, it has been linked to the *BRAF* V600E mutation. Despite the rapid growth pattern, it usually has a favorable prognosis after neurosurgical excision. The presence of this extremely rare, genetically linked tumor, and its combination with Down’s syndrome, the most common human genetic defect, makes this a very novel clinical presentation. It also raises a very research-worthy question of an undiscovered link between these two genetic disorders.

**Case presentation:**

In this case, we report a 1-year-old Pakistani origin male child with Down’s syndrome, who presented with progressive macrocephaly and developmental regression over the last 2 months. He was unable to sit by himself, and had lost his handgrip bilaterally. Down’s Syndrome was diagnosed soon after birth, based on typical facial features and presence of palmar crease, and later confirmed karyotypically for Trisomy 21. Upon presentation, initial blood tests did not show any abnormality. Magnetic resonance imaging of the brain was done, and showed a mixed intensity cystic mass with solid dural component posteriorly in the right parieto temporo occipital region. Craniotomy was performed, and about 85% of the tumor mass was excised. Histological examination and immunochemistry confirmed the suspected radiological diagnosis of desmoplastic infantile astrocytoma. After surgical excision, our patient gradually reacquired his previously regressed developmental milestones. Unfortunately, the remaining mass, which could not be excised due to its attachment to the highly vascular dura mater, showed regrowth on repeat brain magnetic resonance imaging. As his parents did not consent to further surgery, chemotherapy was offered as the next treatment option to prevent tumor regrowth.

**Conclusions:**

This case report highlights the need for more case data and research to understand desmoplastic infantile astrocytoma, and their genetic correlation with Down’s syndrome. From a clinical standpoint, since desmoplastic infantile astrocytoma has a good postresection prognosis in a majority of early-diagnosed clinical cases, pediatricians, radiologists, and pathologists should consider desmoplastic infantile astrocytoma in their initial differential diagnosis in Down’s syndrome patients with macrocephaly and developmental regression during the first 2 years of life.

## Introduction

Desmoplastic infantile astrocytoma (DIA) and desmoplastic infantile ganglioglioma (DIG) are classified as glioneuronal and neuronal tumors by the 2021 World Health Organization (WHO) classification of central nervous system (CNS) tumors [1]. They are rare and often benign brain tumors linked to *BRAF* V600E mutations [2]. Despite their aggressive radiological appearance, they have a good postresection prognosis [3]. Desmoplastic infantile astrocytoma/ganglioglioma (DIAGA) mostly occur in children < 2 years old; however, 23% occur in children > 2 years old [4]. They are characterized histologically by a prominent desmoplastic stroma with a variable neuroepithelial component consisting of neoplastic astrocytes and ganglion cells [4]. They are exclusively supratentorial, with a large voluminous size, and are partially cystic [5]. Total surgical removal is sufficient for the treatment of these tumors, and no chemotherapy or radiotherapy is indicated if complete resection is achieved. Prognosis is usually good, with ~10% reported mortality in recurrent cases [6].

Here, we present a case of 1-year-old male with Down’s syndrome, who came to our clinic with a rapid increase in head size, and regression of previously achieved milestones. Radiological imaging showed a large mass in the right parietal, temporal, and occipital regions, suggestive of DIAGA. The tumor was removed, and histology showed classic features of DIA. This case report extends the currently limited research and education related to this very rare pediatric CNS tumor. In addition, this is the first documented case of DIAGA in a patient with Downs’ syndrome, which begs consideration of an undiscovered correlation between these two clinical disorders.

## Case presentation

A 1-year old Pakistani origin male child with Down’s syndrome, presented with gradual enlargement of the head, and developmental regression over the last few months. He was born at 38 weeks via vaginal delivery after an uncomplicated pregnancy to nonconsanguineous parents (mother 33 years old, and father 38 years old). His birth weight was 2.95 kg, length was 52 cm, and head circumference was 35 cm. His Appearance, Pulse, Grimace, Activity, and Respiration (APGAR) score was 7 at 1 minute, and 10 at 5 minutes. Down’s syndrome was diagnosed soon after birth, based on typical facial features and presence of palmar crease, and later confirmed karyotypically for Trisomy 21. His two siblings, aged 6 and 4 years, were alive and healthy. Further social and family history was unremarkable. From a growth standpoint, at birth, he was 10th percentile for weight and 25th percentile for length. At both 6 months and 1 year, he was 25th percentile for weight and 50th percentile for length. His head circumference was 50th percentile at birth, at 6 months it was at 97th percentile, and at 1 year when he presented to the clinic, it was 20 standard deviations (SD) above the 99th percentile. From a growth milestone standpoint, at 9 months, he smiled and laughed at “peek-a-boo,” lifted arms up to be picked up, crawled, and sat up without support. However, at 12 months, his parents noticed that he cried more, stopped responding to his name, and preferred to lay flat rather than crawl or sit. He was also less vocal and less interested in toys.

**Fig. 1:**
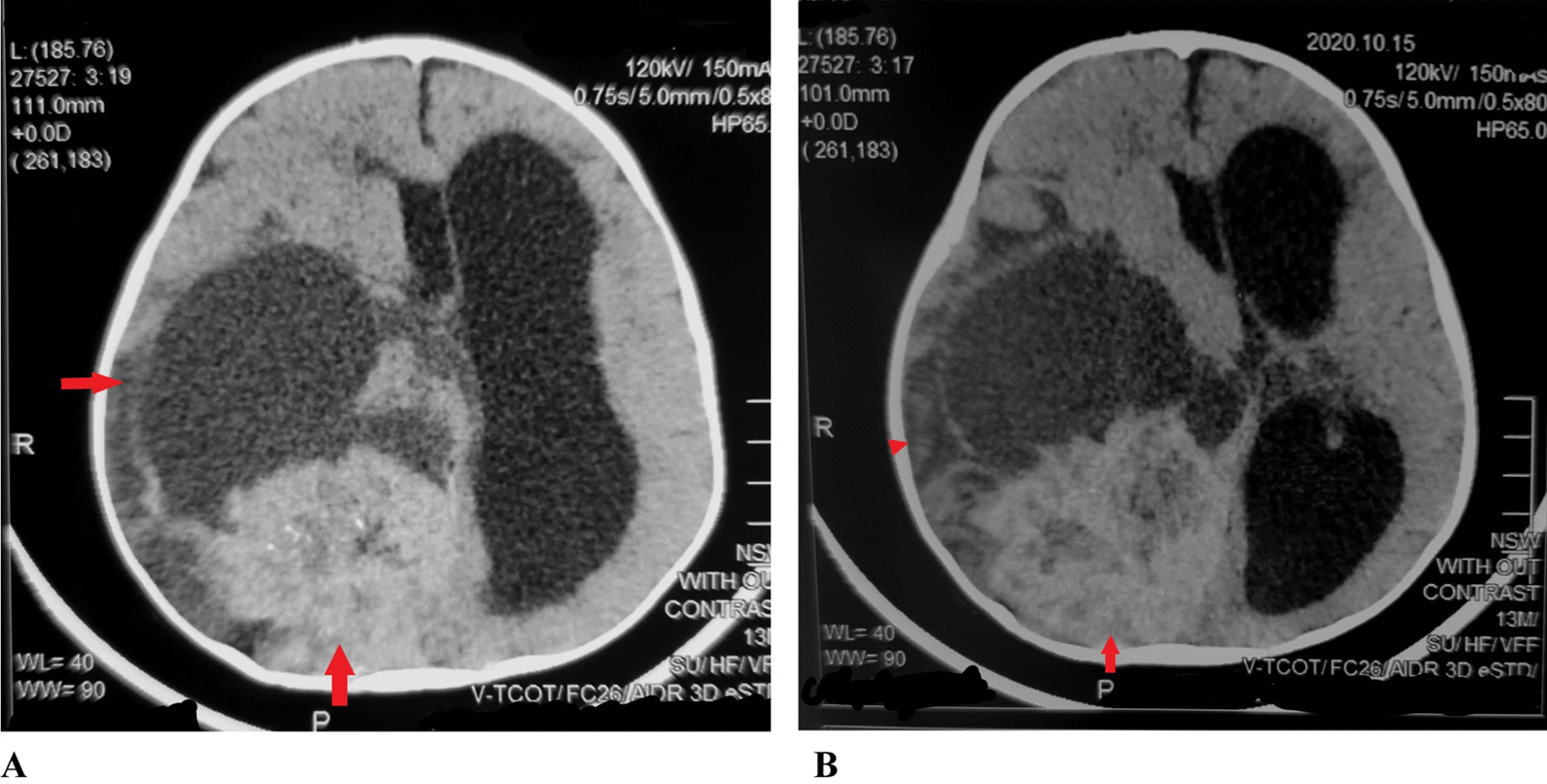
**A**, **B** Pre-operative CT without contrast: Axial images show a mixed density heterogeneously enhancing mass in the Right posterior parieto temporo occipital locations (red arrows)

**Fig. 2:**
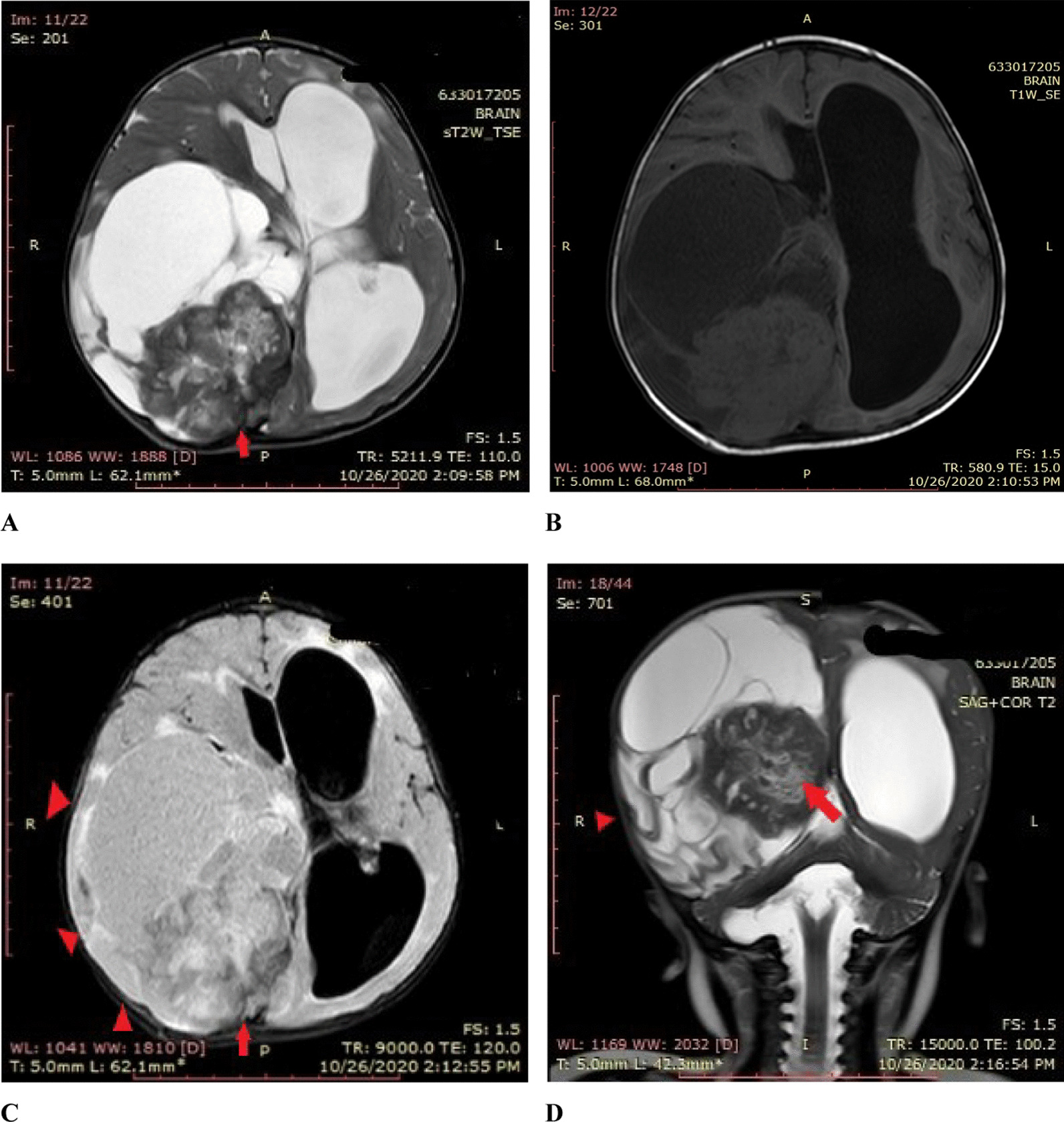
Pre-operative MRI without contrast. **A–C** Axial T2 W/T1W/FLAIR images show a heterogeneous intensity mass (red arrow shows solid component), (red arrowhead shows the cystic component) in the right parieto temporo occipital location compressing ipsilateral lateral ventricle causing a mild midline shift. **D** Coronal T2W images show a heterogeneous signal intensity mixed solid and cystic mass in right parieto temporo occipital location compressing the ipsilateral right lateral ventricle with obstructive hydrocephalus

**Fig. 3:**
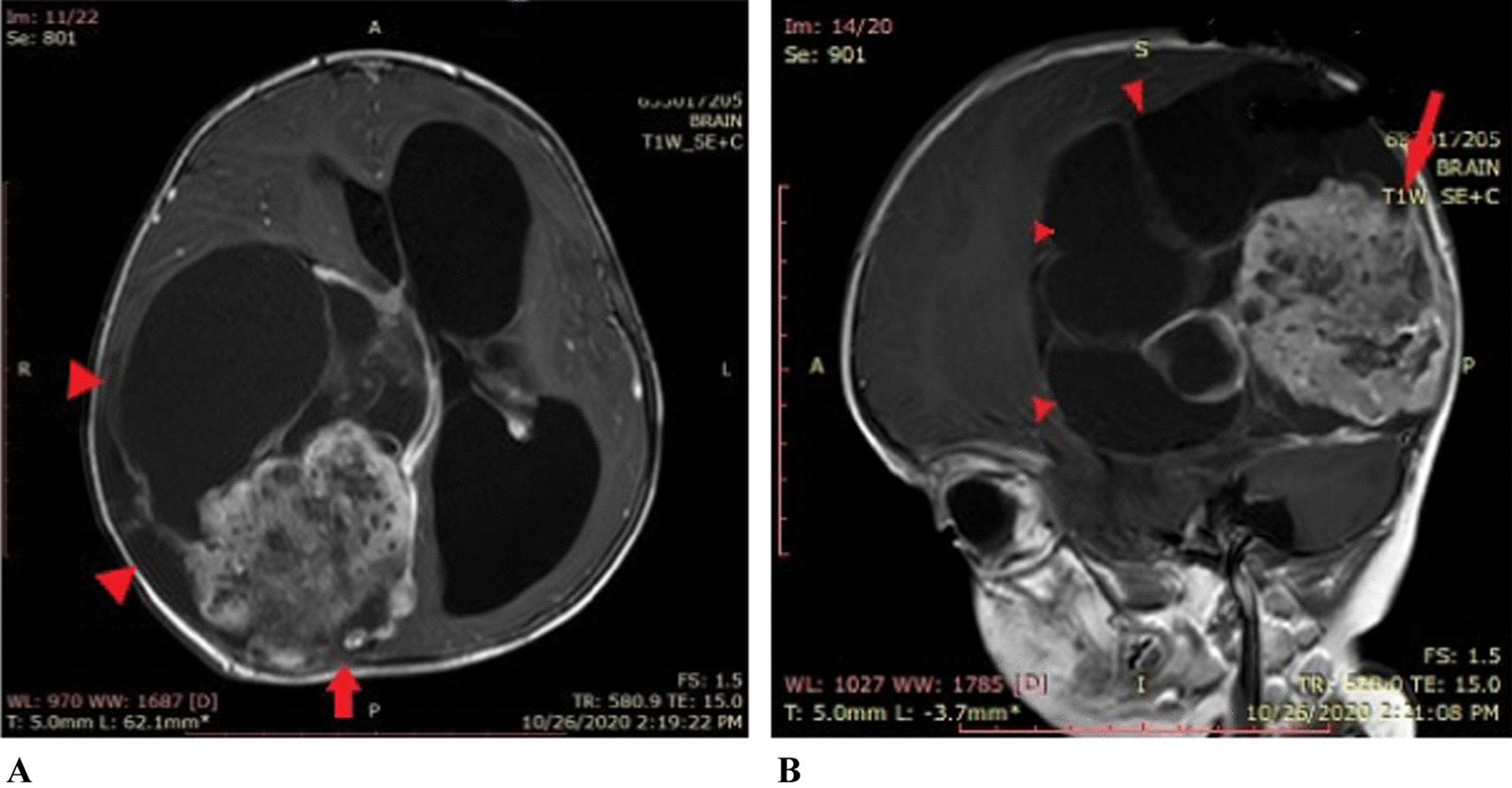
**A**, **B** Pre-operative MRI post-contrast: Axial and sagittal images show a heterogeneously enhancing predominantly cystic mass (red arrow heads) in the right parietal occipital location with a solid heterogeneously enhancing component attached to the dura (red arrow)

On physical examination, he appeared to have macrocephaly, along with signs of Down’s syndrome including flat nasal bridge, upward slanting palpebral fissures, epicanthic folds, and simian crease. He appeared to be mildly irritable. Cardiac, respiratory, abdominal, genitourinary, and integumentary examinations were unremarkable. Neurological examination showed intact cranial nerves and sensations to noxious touch. However, there was generalized muscular hypotonia with muscle wasting, with −2 bilateral knee and ankle reflexes, and bilateral upgoing plantars. His initial blood tests did not show any abnormality. His hemoglobin (Hb) was 11.5 g/dL and white blood count (WBC) was 13 × 10^9^/L with normal differential. His liver and renal functions were within normal limits. Prothrombin time and activated partial thromboplastin time were also within normal limits. Hepatitis B and C serology were negative. Urinalysis and chest X-ray also did not show any abnormality. He was admitted, and a computed tomography (CT) scan was performed, which showed a large cystic tumor in the right parieto temporo occipital regions (Fig. 1). Subsequent magnetic resonance imaging (MRI) of the brain with contrast showed a 10.9 × 11.4 × 8.9 cm, heterogeneously enhancing, mixed solid and cystic supratentorial mass, with surrounding vasogenic edema in the right parieto temporo occipital lobe. The solid component was dural based, measuring 6.4 × 6.0 cm, showing heterogeneous intensity on T2W imaging with marked enhancement on post-contrast images. There was a midline shift towards the left, with compression on the ipsilateral right lateral ventricle. Moderate obstructive hydrocephalus was also noted (Figs. 2, 3).

Two weeks later, a right partial craniotomy was performed, and the cystic component of the tumor was completely excised. Removal of the posterior dural-based solid mass was omitted due its attachment to the highly vascular dura mater. Postoperative course was uneventful.

**Fig. 4:**
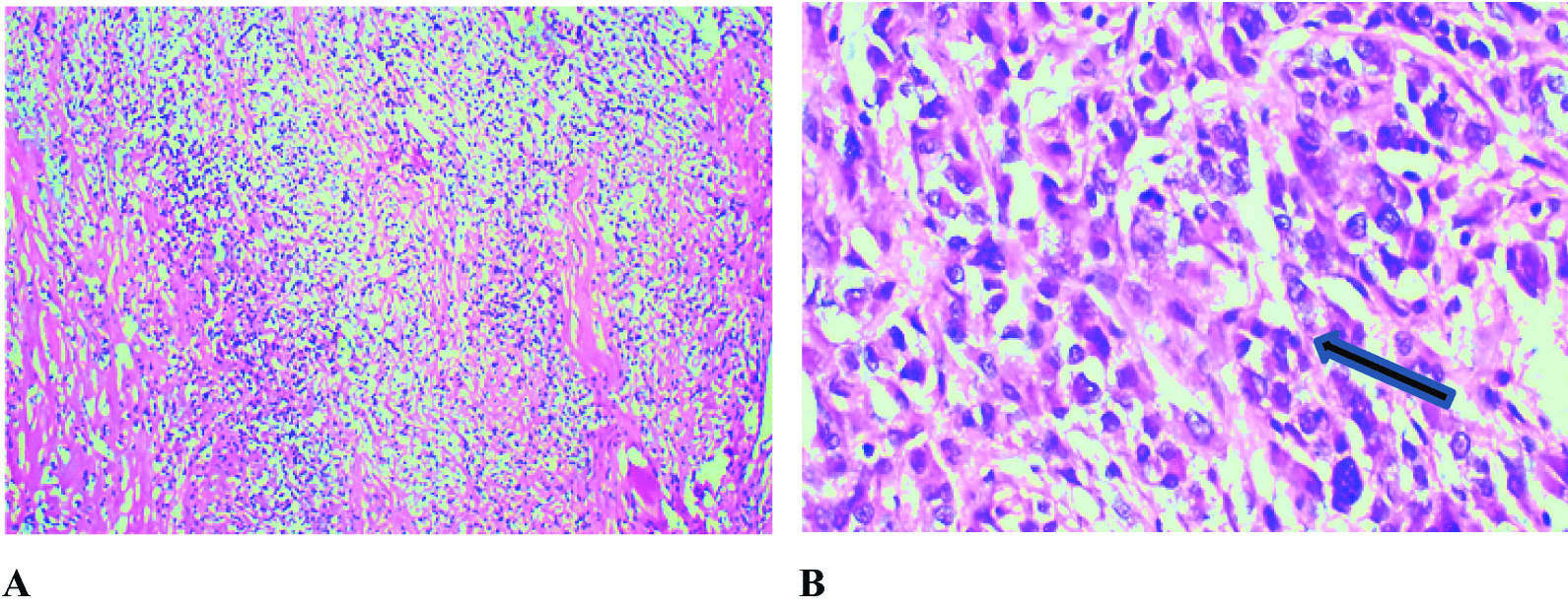
**A** Low-power view highlighting collagenized septa with small cell population having high N/C ratio. **B** High-power view showing scattered cellular foci with pleomorphism and increased mitotic activity (blue/black arrow)

**Fig. 5:**
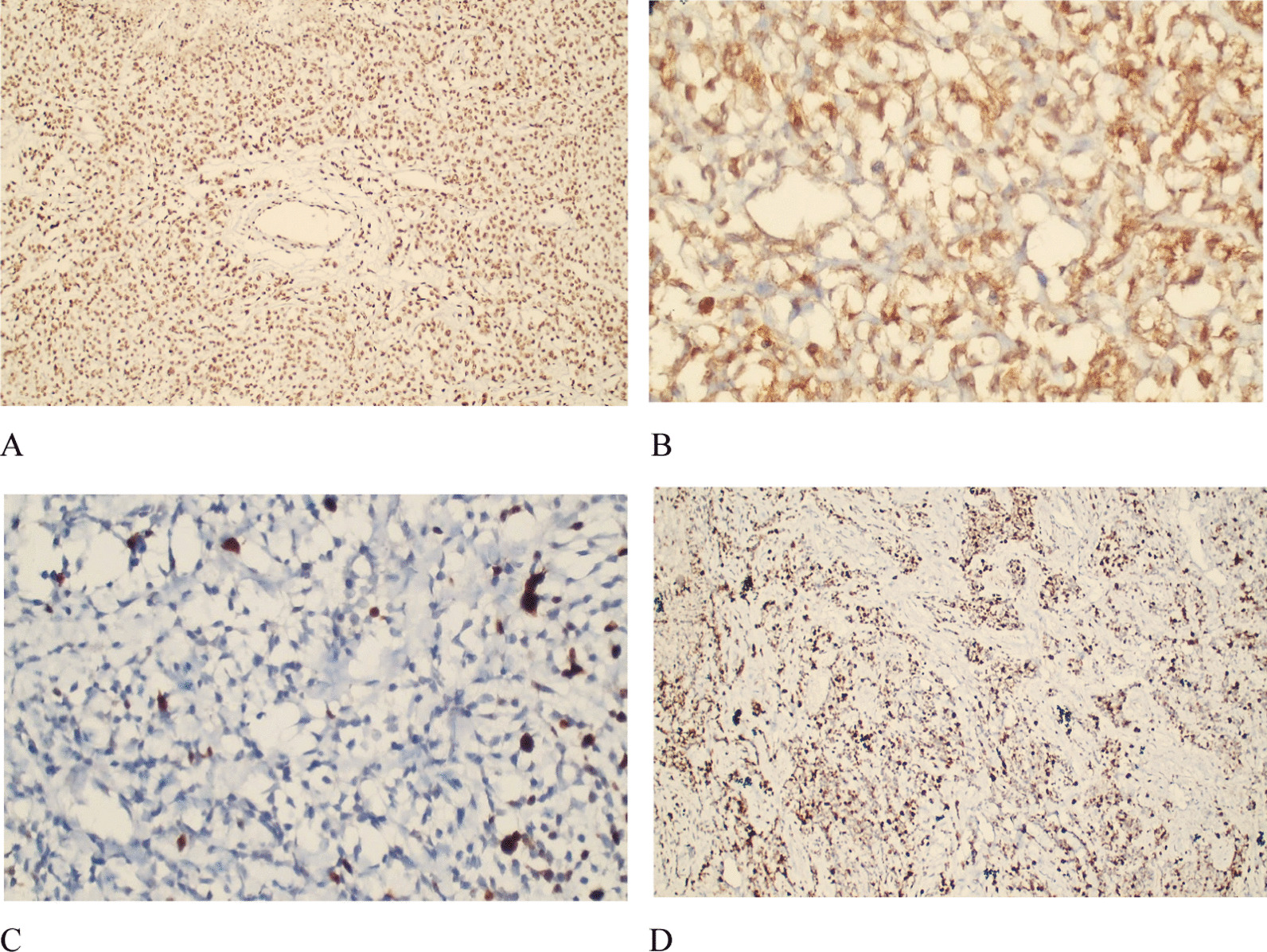
Immunohistochemistry. **A** INI-1 strongly positive in all tumor cells. **B** GFAP positivity observed in neoplastic cells. **C** Ki-67 low in majority of the tumor. **D** High Ki-67 index in areas with increased cellularity

The tumor specimen was sent for histopathological examination, which revealed low cellularity with intervening bands of fibrous tissue and collagen fibers. Neoplastic cells had a high nuclear/cytoplasmic (N/C) ratio with hyperchromatic nuclei. Some areas showed hypercellular pleomorphism, necrosis, and microvascular proliferation. Some cells with rhabdoid morphology were also present with abnormal mitosis (Fig. 4). Staining was performed for integrase interactor 1 (INI-1), glial fibrillary acidic protein (GFAP), desmin, creatine kinase (CK), synaptophysin, and Ki-67. INI-1 came out strongly positive, ruling out atypical teratoid and rhabdoid tumors. A large proportion of tumor cells were positive for GFAP (Fig. 5) and negative for synaptophysin, desmin, and CK. Ki-67 index was low in the majority of the tumor. However, areas with increased cellularity had raised Ki-67 to 50%. Morphology and immunohistochemistry confirmed the diagnosis of DIA with aggressive histological features, which correlated with clinical and radiological findings. Further molecular studies were not done.

**Fig. 6:**
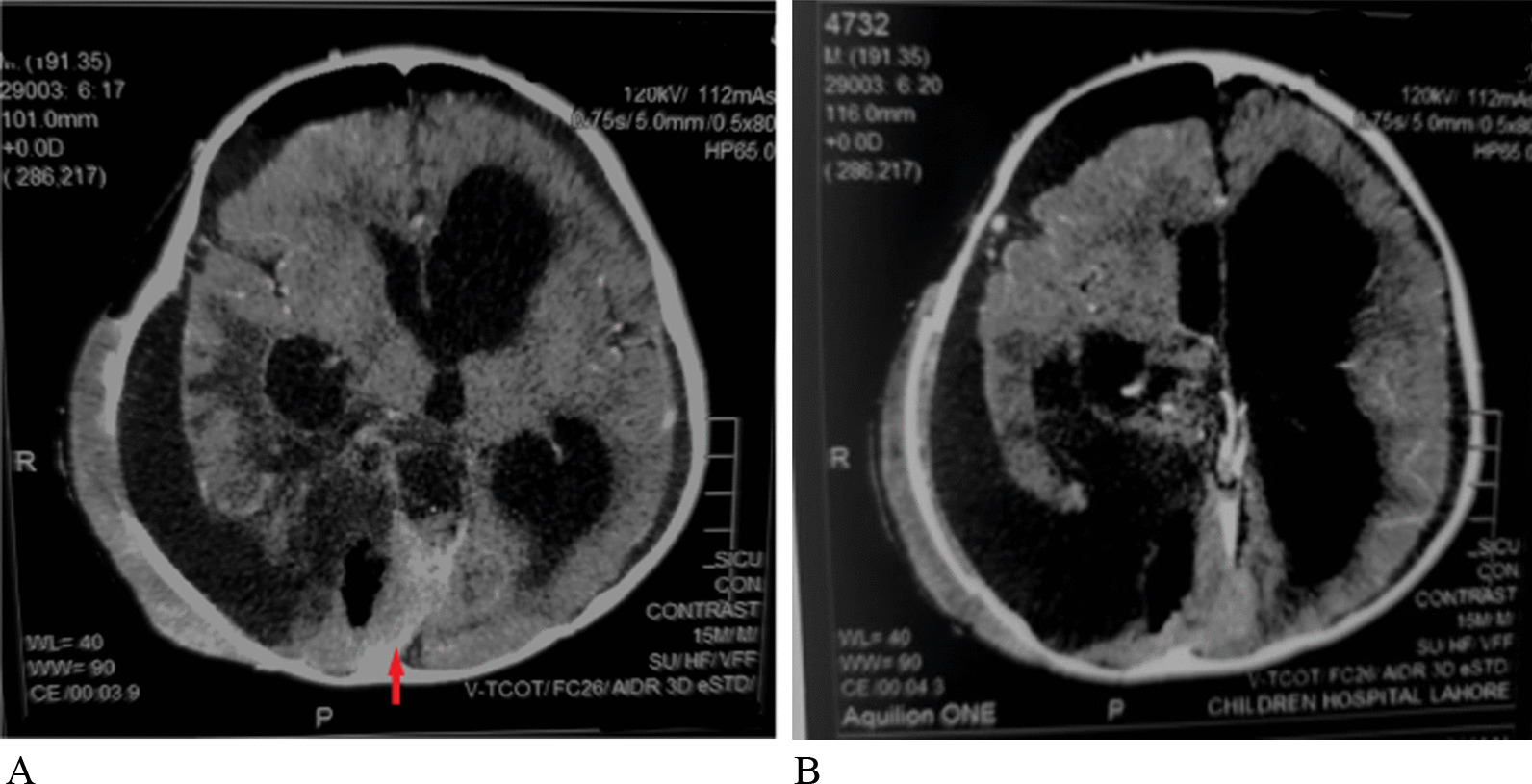
**A**, **B** Postoperative CT without contrast: A major bulk of the tumor had been removed with a small enhancing dural residual mass (2.9 × 1.3 cm on axial images) adherent to posterior interhemispheric fissure (red arrows).

A postoperative CT was done that showed removal of the major bulk of the tumor. A residual mass (3.1 × 1.3 cm on axial images) was seen adherent to the dura of posterior interhemispheric fissure (Fig. 6). The patient was subsequently discharged home. At follow-up at 6 weeks, his mother reported gradual functional improvement, along with recovery of motor milestones evidenced by his ability to sit with support, and return of moderate hand grip.

**Fig. 7:**
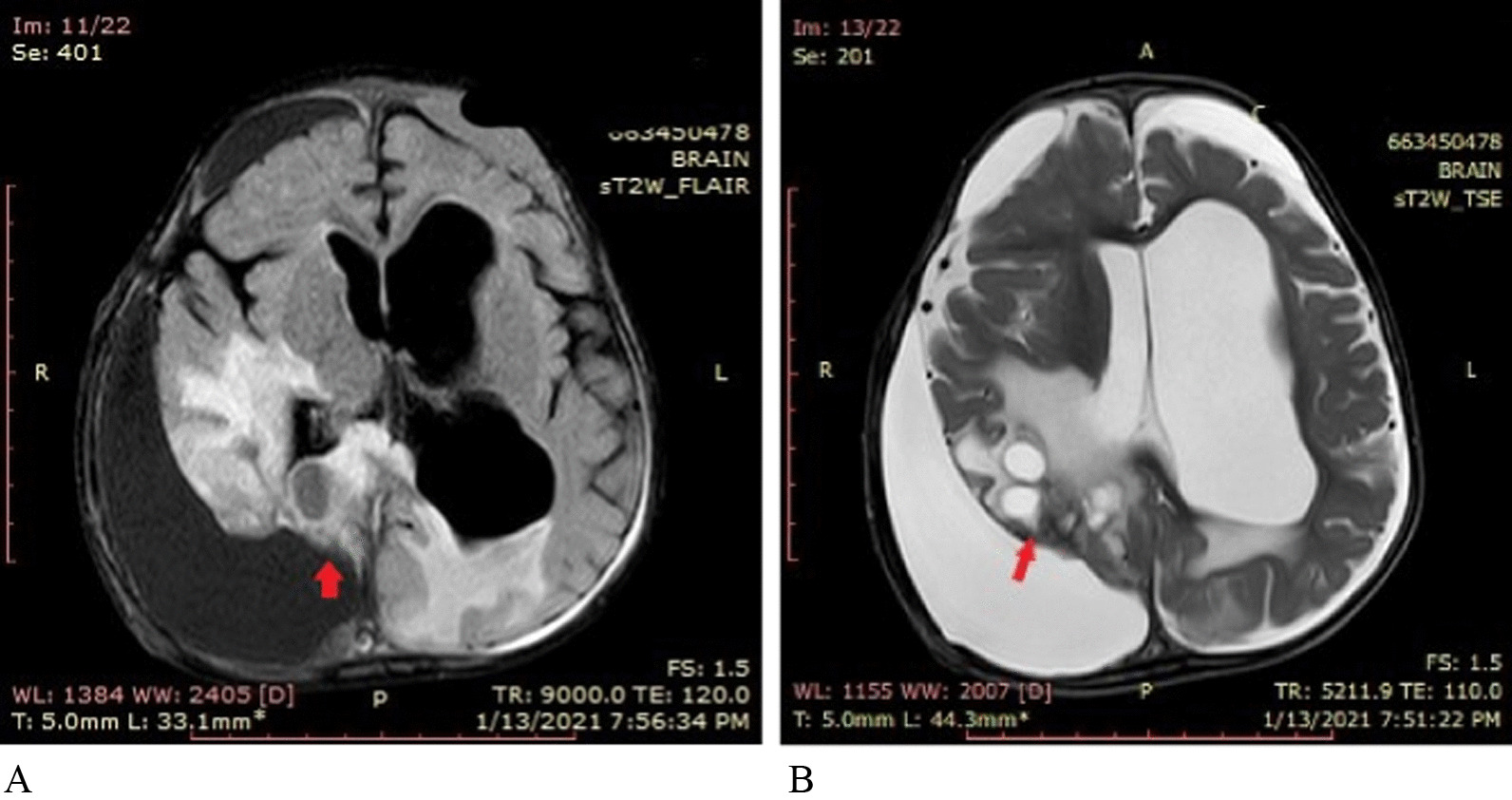
Two months postoperative MRI without contrast. **A** T1 images show an iso to hypo mass, **B **T2/FLAIR images show a mixed intensity image in a right occipital location adherent to the dura with moderate subdural collection

**Fig. 8:**
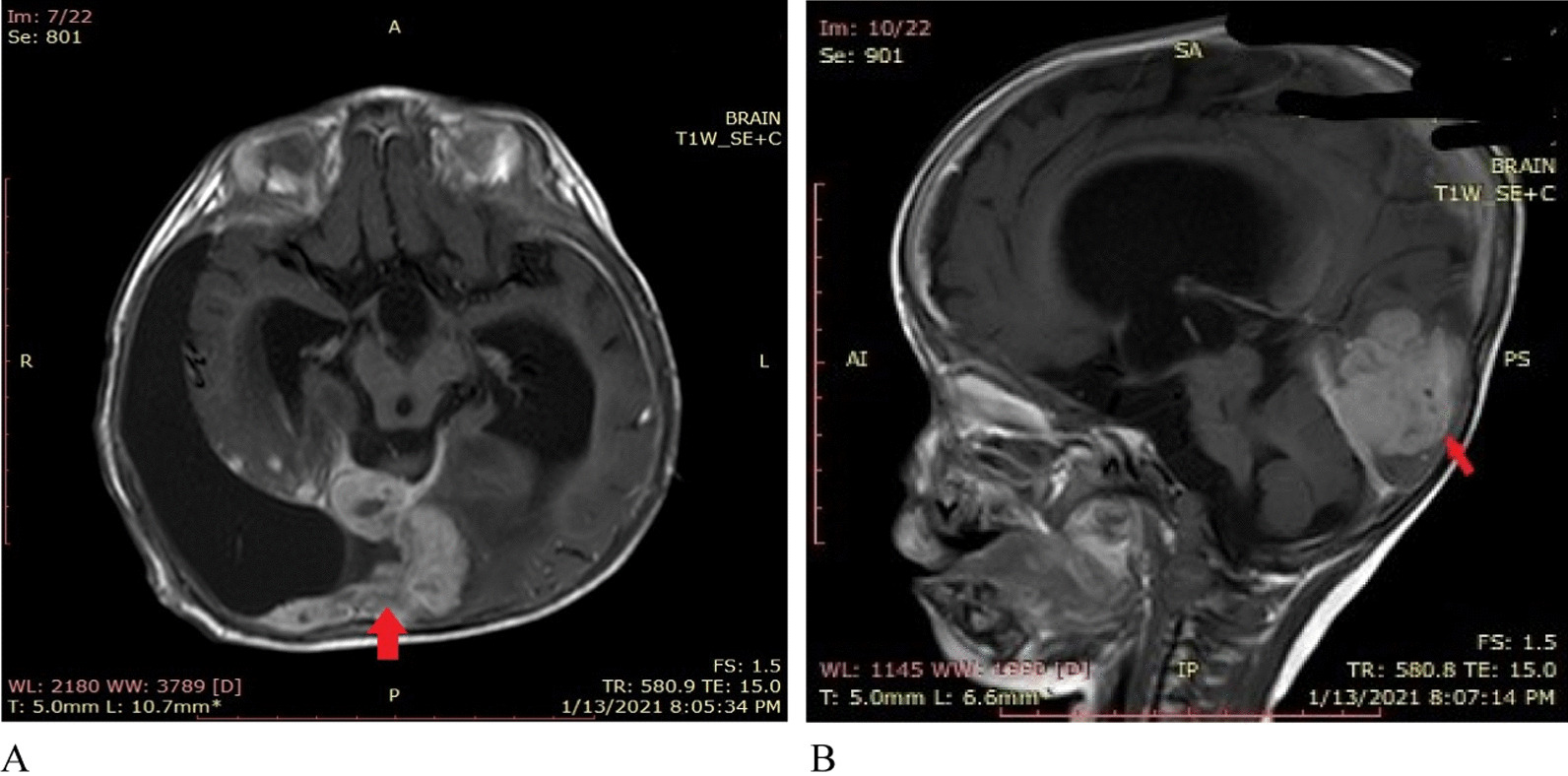
**A**, **B** Two months postoperative MRI with contrast. Axial and sagittal images show a homogeneously enhancing dural based mass (4.2 × 2.6 cm) (red arrow) adherent to tentorium cerebelli and posterior interhemispheric fissure

A 2 months postoperative, brain MRI with contrast showed a comparatively enlarged 4.2 × 2.6 × 4.1 cm homogeneously enhancing dural-based residual supratentorial mass along the right occipital location. This mass was adherent to the posterior interhemispheric fissure, as well as the tentorium cerebelli (Figs. 7, 8). Keeping in view this extremely rare presentation of high-grade DIA with growing residual mass adherent to the dura, prospective treatment options were discussed by the multidisciplinary tumor board. With recurrence after the initial surgery, the parents were nonconsenting for further surgical intervention, and chose chemotherapy in the hope of controlling further tumor growth and halting subsequent functional decline.

Of note, although Institutional Review Board (IRB) approval was not needed for this case report, detailed written informed consent was obtained in English from the parents of the patient (who were fluent in the language) for the use of clinical data, imaging, and histology slides for publication purposes.

## Discussion

In summary, this is a case of DIA in a 1-year-old patient with Downs’s syndrome. DIA presented at the age of 1 year, with a relatively rapid increase in head size and regression of developmental milestones, along with a large mixed density heterogeneously enhancing brain mass, with both solid and cystic elements on imaging. Histology/immunohistochemistry showed collagenized septa with a small cell population with a high N/C ratio, pleomorphism, increased mitotic activity, strong INI-positivity, GFAP positivity, and a high Ki-67 index in cellular components of the tumor that further confirmed the diagnosis. Although some rare cases of this tumor have been reported in the literature, none have been found in a patient with Down’s syndrome. This makes this case very unique for an undiscovered link between Trisomy-21 and *BRAF* V600E mutations, which can be further explored through future laboratory genetic studies. But, most importantly, such linkages can help develop new targeted therapies for such mutations that cause such gliomas, rather than current empirical surgical and chemotherapeutic regimens that have lower efficacy for other related and more common CNS gliomas, with much worse outcomes and prognosis.

Desmoplastic supratentorial neuroepithelial tumors of infancy are a rare but distinctive group of tumors that affect children less than 2 years of age [6]. These tumors were previously named DIA and DIG, based on immunohistochemistry. DIG and DIA are classified as glioneuronal and neuronal tumors in the 2021 WHO classification of CNS tumors [1]. Recent genetic studies have linked them to the *BRAF* V600E gene mutation [2]. Frontal and parietal lobes are mostly affected by this tumor [5]. The median age for diagnosis is 5–6 months [7], while the age range is 1–24 months, with a male to female ratio of 1.7:1 [8]. Common presenting complaints include macrocephaly, seizures, and psychomotor delay [3].

CT and MRI usually show a large superficial cerebral mass with both solid and cystic components [5]. The major bulk of the tumor contains large cysts with minimal surrounding vasogenic edema. The cysts are deeply placed, while the solid part is usually peripheral. On MRI, the cystic part of the tumor follows CSF intensities on T1W/T2W pulse sequences [5]. It is hyperintense on FLAIR imaging, and rarely enhances on post-contrast imaging, while the solid portion is isointense on T1W images and hypointense/mixed intensity on T2W/FLAIR images [5]. Post-contrast MRI usually depict enhancement of the solid part of the tumor. It is rare to detect calcification in DIA [9].

Histology usually shows low cellularity with intervening bands of fibrous tissue and collagen fibers. Neoplastic cells usually have a high N/C ratio with hyperchromatic nuclei. Hypercellular pleomorphism, necrosis, and microvascular proliferation are often seen with abnormal mitosis [10]. Staining is strongly positive for INI-1 and GFAP, and negative for CK, synaptophysin, and desmin. The Ki-67 index is usually high in these often aggressive tumors [10].

Going back to our clinical case, after refusing more surgical treatments, the parents chose to try chemotherapy as the next line of treatment. This case was discussed by the institutional tumor board, and it was decided to proceed with a combination regimen of vincristine and carboplatin, based on the tumor histology and literature support. The patient received three cycles of this treatment before developing pancytopenia. He was given blood transfusions, and granulocyte colony-stimulating factor (G-CSF) treatment, but soon developed a lower respiratory tract infection with respiratory failure. He was transferred to the intensive care unit (ICU) where he was intubated and mechanically ventilated, but was unable to come off the ventilator. He later had a tracheostomy, and a G-tube placed. Unfortunately, he continued to have more respiratory complications, and died of sepsis related complications after 6 weeks in the ICU, and 6 months after his initial diagnosis.

Most case reports of DIA have reported a favorable prognosis. Surgery is often curative [3]. Residual DIAs have been described as spontaneously regressing after surgery, likely attributed to loss of vascularity after surgery. Due to the large size and adhesiveness to the brain, complete excision is often not possible [3]. In such situations, chemotherapy and then radiotherapy are added to the treatment regimen [11, 12]. Radiotherapy is recommended as a palliative treatment after chemotherapy failure and in patients > 5 years. Out of a total of 107 cases in the literature, only 76 had reported initial surgical outcomes. Out of these 76 cases, 47 had a gross total resection, 26 had a subtotal resection, and 3 had only an initial biopsy. Most patients (60.7%) had a clinically benign course after initial surgical resection, while the remainder required further treatment (reresection, chemotherapy, and/or radiotherapy) [13].

In our case, the patient presented within the typical spectrum of the disease symptoms and other typical demographics. The standout point here was the presence of Down’s syndrome as a prior comorbidity, which makes it the first case of a DIAGA tumor in a Trisomy 21 patient. With a genetic basis in *BRAF* V600E mutation, this leads to the question of an undiscovered association of Trisomy 21 with *BRAF* mutations. In addition, from a clinical standpoint, this is a very treatable tumor, and early imaging and surgery can provide complete cure, and restore good physical function in most cases. This recovery and cure aspect makes it a rare yet very important differential for pediatricians, radiologists, and pathologists who deal with this patient population.

## Conclusion

DIAs are rare brain tumors that can occur throughout childhood, and should therefore be included in the differential diagnosis in children with brain tumors. Here, we present the first case of DIA in an infant with Down’s syndrome, who presented with macrocephaly and developmental regression. Radiological images showed a mixed solid and cystic, space-occupying lesion for which surgery was done with substantial functional recovery. Based on clinical, radiological, and histopathological findings, a diagnosis of DIA was made. This case adds to the currently limited spectrum of research and education related to this very rare pediatric CNS tumor. In addition, this is the first documented case of DIAGA in a patient with Downs’ syndrome, that begs consideration of an undiscovered correlation between these two clinical disorders, which should be further scientifically examined with additional future research studies.

## Data Availability

We have original data on file available for review, if requested.

## Abbreviations

DS: Down’s Syndrome
WHO: World Health Organization
DIA: Desmoplastic infantile astrocytoma
DIG: Desmoplastic infantile ganglioglioma
DIAGA: Desmoplastic infantile astrocytoma/ganglioglioma
INI-1: Integrase interactor 1
GFAP: Glial fibrillary acidic protein
CK: Creatine kinase
N/C: Nuclear/cytoplasmic ratio
